# Non coding RNA and brain

**DOI:** 10.1186/1471-2202-7-S1-S5

**Published:** 2006-10-30

**Authors:** Carlo Presutti, Jessica Rosati, Sara Vincenti, Sergio Nasi

**Affiliations:** 1 Dipartimento di Genetica e Biologia Molecolare, Moro 5, 00185 Roma, Italy; 2 IBPM CNR, Università La Sapienza, P.le A. Moro 5, 00185 Roma, Italy

## Abstract

Small non coding RNAs are a group of very different RNA molecules, present in virtually all cells, with a wide spectrum of regulatory functions which include RNA modification and regulation of protein synthesis. They have been isolated and characterized in all organisms and tissues, from Archaeobacteria to mammals. In mammalian brain there are a number of these small molecules, which are involved in neuronal differentiation as well as, possibly, in learning and memory. In this manuscript, we analyze the present knowledge about the function of the most important groups of small non-coding RNA present in brain: small nucleolar RNAs, small cytoplasmic RNAs, and microRNAs. The last ones, in particular, appear to be critical for dictating neuronal cell identity during development and to play an important role in neurite growth, synaptic development and neuronal plasticity.

## Introduction

In recent years it has become more and more clear that RNA cannot be any longer classified as ribosomal, messenger and transfer RNA (rRNA, mRNA, tRNA); instead a number of different RNA molecules have been characterized, which are involved in a variety of cellular regulatory processes on their own or as a part of ribonucleoprotein particles (RNPs) [1]. Small nuclear RNAs (snRNAs), small nucleolar RNAs (snoRNAs), microRNA (miRNAs), just to name a few, have revolutionized our view of cellular molecular machineries. It is now predicted that as much as 40–50% of mammalian mRNAs could be regulated at the translational level by miRNAs and that 50–60% of mammalian pre-mRNAs could be alternatively spliced, by a process critically involving snRNAs, to form a huge number of different proteins.

A number of small non-coding RNAs have been detected in mammalian brain; among them, some appear to be brain specific. They have been related to brain development, neuronal differentiation and complex functions such as learning and memory; some have also been implicated in neuronal degeneration and mental disorders.

## Small nucleolar RNA

**Figure 1:**
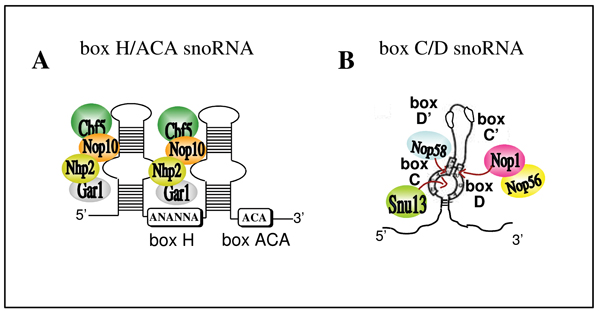
**Schematic secondary structure of the two classes of snoRNAs**. A) H/ACA box snoRNAs fold into "hairpin-hinge-hairpin-tail" structures and contain two short sequences called boxes H and ACA, as indicated in the white boxes. The hairpins are interrupted by an internal loop that forms the pseudouridylation pocket. A set of conserved proteins, Nhp2, Gar1, Cbf5 and Nop10, recognize and bind to this structure. B) C/D box snoRNAs show conserved boxes termed C (UGAUGA) and D (CUGA), positioned near their 5' and 3' termini. Sometimes two additional, less conserved, boxes called C' and D' are present. C and D boxes form a characteristic secondary structure: the K-turn motif. Four evolutionary conserved proteins are associated to it: Snu13 (15.5K in human), that recognizes the internal loop [111], Nop58 and Nop1 (Fibrillarin in human) that cross-link to the U in box C and the U in box D, respectively [112]. In yeast, Nop58 and Snu13 are necessary for the accumulation of all C/D box snoRNAs [113], Nop1 is essential for the production of a subset of box C/D snoRNAs, and Nop56p is not essential for snoRNA accumulation [114].

All eukaryotic cells contain a large number of small ribonucleoprotein particles (snoRNPs) active in rRNA processing, modification and ribosome assembly. These particles are formed by a single snoRNA molecule and a set of proteins. SnoRNAs, and snoRNPs, can be of two different classes: "C/D box", containing conserved sequences named boxes C and D, and "H/ACA box", again containing conserved sequences with those names (Figure 1). Different proteins bind to the two classes, forming the ribonucleic particles snoRNPs [2]. These snoRNPs, more specifically their snoRNA component, target specific sequences on pre-rRNA for nucleotide modification: box C/D snoRNAs cause 2'-O methylation [3] while the box H/ACA ones give rise to pseudouridylation [4]. In both these processes, the modification reactions are guided by the joining of complementary regions, extended for 10–21 nt, between the rRNA and the snoRNA. The same reaction of site specific nucleotide modification occurs on rRNA, tRNA, snRNA and, most probably, mRNA. rRNA and tRNA nucleotide modifications are thought to improve translation efficiency while modification of other RNA molecules probably affects their folding.

Most mammalian snoRNAs are ubiquitous, but some are expressed exclusively in brain. This suggests that these molecules are not needed for normal housekeeping, but are important for brain function. Six C/D box and one H/ACA box RNAs have been isolated from mouse brain. MBII-13 and MBII-78 are encoded by single copy genes while MBI-36 (H/ACA), MBII-48, MBII-49, MBII52 and MBII85 are encoded by multiple copies [5]. Human homologues of these snoRNAs are also highly expressed in brain while a couple of them, HBII-13 and HBII-85, are also found in other tissues [6]. In situ hybridizations revealed that MBI-36, MBII-48, MBII52 and MBII85 are not expressed uniformly in brain. They are concentrated in hippocampus and amygdala, two areas very important for brain functions such as spatial learning and fear conditioning. Furthermore, inside the hippocampus, a difference exists between dorsal and ventral regions: MBI-36, MBII-48, MBII52 have a higher expression in ventral with respect to dorsal hippocampus, suggesting that these snoRNAs play a differential role [7].

The function and molecular mechanisms of brain specific snoRNAs remain unknown. They have no complementarity to rRNA, so they are probably not involved in rRNA processing or modification. Their peculiar hippocampal expression pattern, however, raises the intriguing possibility that they are involved in memory consolidation. In fact, ventral hippocampus has been demonstrated to be very important in contextual conditioning, a form of association of stimuli with the context [8]. While MBI-36 expression does not change in contextual memory consolidation, MBII-48 and MBII52 expression is transiently regulated, being lower for the first and higher for the second. Furthermore, this variation is strictly associated with contextual conditioning and it is not induced by a different kind of stimulation [7]. As stated above, the molecular mechanism of action of these RNAs remains obscure. However, they have a complementarity to mRNAs for proteins involved in memory processes like for instance 5-HT_2C _mRNA, coding for the serotonin 2C receptor. This receptor is involved in memory consolidation [9,10]; editing and alternative splicing of its mRNA have been associated with mental disorders such as schizophrenia [11,12]. Human homologues of MBII-13, MBII-52 and MBII-85 map on chromosome 15q11-13 region, which is associated with Prader-Willi syndrome (PWS), a neuro-behavioural disorder leading to mental retardation [13,14]. Later studies restricted the PWS locus to a small region containing only the HBII-85 gene, indicating a strong association of this snoRNA with the disorder [15].

## Small cytoplasmic RNA

Brain cytoplasmic RNAs were discovered 20 years ago and are one of the first examples of active genes derived from retro-transposition [16,17]. BC1, in rodents, and BC200, in primates, have similar expression patterns and probably play similar roles in neuronal cells.

BC1 is encoded by the mouse *bc1 *gene located on chromosome 7; it is transcribed by RNA polymerase III and derives from a retro-transposition event of tRNA^ala^. This tRNA gene is believed to be at the origin of Short Interpersed Repetitive Elements (SINEs) rodent sequences, which are similar to Alu sequences in primates. Mutations in re-transposed copies of the gene during evolution would have changed the structure of the RNA molecule from a typical tRNA to a complex stem-loop, changing at the same time the functionality of the molecule [18]. The human gene coding for BC200, *bcyrn1*, is also transcribed by RNA polymerase III and is located on chromosome 2. BC200 is structurally similar to BC1 and contains three structural domains: the 5' domain, similar to the Alu repetitive element, a central domain rich in adenines, and the 43 nt long 3' terminal domain [19].

BC1 RNA is found only in neural tissues [20], and is present in widely different amounts in different brain structures: it is highly expressed in olfactory bulb, hippocampus and cortical neurons, while it is absent or present in very low quantity in other brain areas [21]. The cellular localization of BC1 is also peculiar: it is found in axons and concentrated in somato-dendrites [22]. BC200 share the same localization: it is expressed in neurons and transported to dendrites [19]. The intra-cellular localization of BC1 and BC200 suggests their involvement in regulation of translation in dendrites and synapses. In fact, these RNAs bind to fragile × mental retardation protein (FMRP), a protein that inhibits translation of specific mRNAs at synapses [23,24]. BC1 and BC200 can probably guide FMRP to specific targets, as they have regions of complementarity to different mRNAs, also localized at synapses, like Arc, MAP1B and αCaMKII. These mRNAs code for proteins involved in synaptic plasticity phenomena such as long term potentiation (LTP), implicated in learning and memory [25,26]. Furthermore, BC1 and BC200 have been shown to inhibit small ribosomal subunit recruitment on some mRNAs [27] and to associate with the polyA binding protein PABP [28]. BC1 RNA production is regulated during neuronal development and during changes in neuronal activity [29]. BC200 expression seems also to be related to Alzheimer's disease: brains of Alzheimer's patients show a strong reduction of BC200 accumulation, linking these RNAs with neuro-degenerative disorders [30]. Knock-out mice for the *bc1 *gene have been generated. They develop normally, but show defects in activity and an increased anxiety. On the other hand, they do not show defects in learning and memory processes [31], indicating that, if BC1 is involved in these processes, some unknown activity must be present to replace it.

### MicroRNA

**Figure 2:**
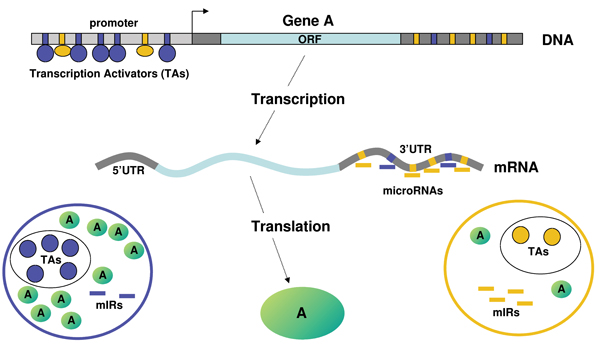
**Combinatorial control of transcription and translation on gene expression**. Protein production depends on the resultant of transcription and translation events, in which transcriptional activators (TAs) and microRNAs (miRs), respectively, are major effectors. Other important post-transcriptional events are not indicated here, but are reviewed in [115]. Cell context specific combinations of TAs and miRs determine protein A biosynthesis:1) The cell on the left (blue context) has a large amount of transcription activators for gene A promoter and a small amount of miR interacting with the 3' UTR of gene A mRNA. As a result of the strong transcription and the low inhibition of translation, protein A is abundant in this cell. 2) On the contrary, in the cell on the right (orange context) the few TAs and the high numbers of 3' UTR interacting miRs determine a low production of protein A.

miRNAs are small RNA molecules involved in regulation of gene expression by sequence specific pairing with mRNA 3' UTRs (untranslated regions) and subsequent inhibition of translation or mRNA degradation [32]. In the recent past, the number as well as the overall importance of these small molecules have increased dramatically: it is now postulated that expression of as much as 50% of mammalian mRNAs could be regulated in such a way [33]. Gene expression control is finely directed by the concerted action of transcription factors and miRNAs (Figure 2). Work in model organisms has given support to the idea that miRNAs have a pivotal function in cell differentiation and the maintenance of cell identity in metazoa, serving as agents for decisions among cells of related fates [34]. Clear examples are found in *C. elegans*. The two miRNAs lsy-6 and miR-237 controls the left/right asymmetry of chemo-sensory receptor expression in taste receptor neurons, through repression of a homeobox transcription factor [35]; miRNA-61, a direct transcriptional target of LIN-12/Notch signaling, determines vulval precursor cells to adopt a spatial pattern [36]. In zebrafish, miR-430 family miRNAs are required for neurogenesis. In *Drosophila*, the bantam miRNA regulates cell proliferation and death by targeting the pro-apoptotic gene *hid *[37]. Only a few targets have been validated in mammalian systems, and the role of miRNAs is less clear [38]. The current view is that, likewise the situation in model organisms, mammalian miRNAs are involved in restricting the fate of progenitor cells. This task is particularly important for the nervous system, in which a vast variety of cell types is derived from a single cell lineage. The role of miRNAs continues beyond development, as these molecules operate in the adult brain as well, where they may function in the local regulation of mRNA translation, playing an important role in axon guidance, synaptic development and neuronal plasticity.

### MicroRNA biogenesis

Biogenesis of miRNAs is a multi-step process beginning with transcription by RNA polymerase II of a primary transcript, called pri-miRNA. This precursor associates inside the nucleus with other factors, still largely unknown, to form a large complex of 650 kDa that cleaves out a 70 nt long stem-loop precursor RNA (pre-miRNA). The cleaving enzymatic activity resides in an RNAse III type endonuclease called Drosha [39]. The 650 kDa complex also contains a double stranded RNA binding protein encoded by a gene deleted in DiGeorge syndrome [40]. RanGTP exportin 5 transports pre-miRNA to the cytoplasm [41], where another RNAse III, Dicer, cleaves the stem-loop structure to produce 20–22 nucleotide miRNA duplexes [42]. One of the two RNA strands is then included into the RNA-induced silencing complex (RISC) [43]. Which strand is included into RISC is determined by the different thermodynamic stability of the two ends of the duplex. The strand with relatively unstable pairs at the 5' end is thought to remain as a mature miRNA. In case the two ends are equally stable, both strands can be incorporated into RISC and function as a mature miRNA [44]. RISC is again a large complex, in which members of the PIWI/PAZ domain containing proteins (Argonaute, AGO) are the principal components. The fact that different members of this protein family are contained in RISC is responsible for the remarkable heterogeneity among such complexes [45].

In humans, Ago2 (hAgo2) is associated with both siRNA and miRNA, and mediates RNA cleavage targeted by small RNAs; however, other Argonaute subfamily members, such as hAgo1, hAgo3, and hAgo4, do not mediate such RNA cleavage, although all can associate with siRNA and miRNA [46,47].

RISC has multiple functions: it stabilizes the target strand, guides the strand to target mRNAs and, depending on the degree of complementarity between miRNA and target, activates endonucleolytic cleavage of the mRNA, probably when there is a perfect pairing, or translational inhibition, when pairing is not perfect.

### MicroRNA detection

miRNAs are easily detected through a number of techniques, such as Northern blot analysis with miRNA specific probes, sequence-directed cloning and sequencing, primer extension followed by real time PCR, and high throughput oligonucleotide microarrays [48]. Locked nucleotide RNAs (LNAs) are a new class of bicyclic high-affinity RNA analogues containing a furanose ring in the sugar-phosphate backbone which is chemically locked in an N-type (C3'-*endo*) conformation by the introduction of a 2'-*O*, 4'-*C *methylene bridge [49,50]. LNA-modified oligonucleotides exhibit exceptionally high affinity and specificity toward their complementary DNA and RNA target molecules. Consequently, an increase in melting temperature (*T*_m_) of + 1–8°C per introduced LNA monomer against complementary DNA, and of + 2–10°C per monomer against complementary RNA, as compared to unmodified duplexes, have been reported. Aside from being highly efficient as Northern blot probes, LNA-modified oligonucleotides can also be useful for addressing the spatial expression of miRNAs by *in situ *hybridization as well as for expression profiling by spotted microarrays containing different LNA-modified capture probes designed to detect both mature and precursor miRNAs.

Besides being experimentally discovered, the existence of miRNAs can be predicted through sophisticated pattern recognition techniques that use the knowledge derived from a set of known miRNAs in order to train a computer program to identifying potential novel miRNA sequences. The combination of multiple properties with appropriately different weights is required for an authentic detection. Most algorithms depend on evolutionary conservation of miRNA sequences between different species: they receive as input sequences that are homologous in two species, and use various procedures to detect miRNAs that are conserved. Open entry servers provide access to a number of RNA folding and prediction algorithms such as Mfold, which models the folding patterns of conserved RNA sequences [51] or MiRscan [52], which employs the RNAFold algorithm [53] in the Vienna RNA package. These procedures allow excluding many false-positive candidates, but are limited to detecting conserved miRNAs. Unlike other techniques, the PalGrade algorithm [48] allows a selection of miRNA candidates that does not depend on sequence conservation. By integrating this algorithm with microarray experiments, it was possible to detect a large number of miRNAs that seem to be unique to primates: out of 89 novel, validated, human miRNAs, 54 were primate-specific [48]. Altogether, it is estimated that 1–2% of mammalian genomes encodes miRNAs [54,55]. Vertebrate miRNAs can be grouped into families in line with their expression at specific time points. The switch from one family to another is accompanied by a change in the expression profile of a cell and its restriction towards a particular lineage: probably miRNAs help tuning the lineage-specificity of cellular protein levels. It is likely that progenitor-specific miRNAs will also exist. Studies that combined bioinformatic predictions with high throughput experimental methods have suggested that the total number of human miRNAs is at least 800, only 400–500 of which are expected to be conserved [33,48].

### MicroRNA targets

The comprehensive identification of miRNA targets is much more complex, as no high throughput experimental methods are at hand. Predicting mRNAs as miRNA targets is computationally hard, since animal miRNAs are only partly complementary to their target mRNAs, and the significance of the mismatches is ambiguous. Furthermore, the interaction between a miRNA and its target is probably not simply a RNA hybridization reaction since it may be affected by proteins in the RISC complex. Unlike miRNA prediction, there does not exist a large enough group of known targets which can be used as a training set for machine learning. Accordingly, the approach for miRNA target prediction is based on empiric evidence. Different aspects of miRNA binding to its targets serve as the basis for prediction. A feature of central importance is the so called "nucleus" or "seed" region at the 5' end: nucleotides 2–8 in the miRNA, often flanked by adenosines, which do often complement the target very closely and are conserved among families of miRNAs [33,56]. This seed has been shown to be critical, and in some cases sufficient, for miRNAs to suppress their targets. There is also evidence that the 3'end of a miRNA may compensate for insufficient base-pairing of its 5' seed. Popular target prediction algorithms, usually freely accessible via web, are Diana-MicroT [57,58], miRanda [59,60], PicTar [56,61], RNAhybrid [62,63], TargetBoost [64], TargetScan [33,65] and miRNA – Target Gene Prediction at EMBL [66]. Predictions have led to the proposal that a single miRNA can bind to a large number of mRNAs; in flies a single miRNA is predicted to have, on the average, 54 targets, a figure that raises to about 200 in humans [33,56,67]. Studies showed that miRNA function may depend on binding to multiple binding-sites in the same mRNA target [68,69]. It is calculated that 30–40% of all targeted transcripts have more than one anchor sites for single miRNAs. In addition to having multiple targets of a single miRNA, a mRNA can bind to multiple different miRNAs. In some instances, miRNAs have been shown to function in a collaborative manner: when any one of the two let-7 miRNA binding sites on its target lin-41 mRNA in zebrafish is replaced by a miR-221 binding site, then both miRNAs are needed to inhibit this target [68]. The most complete miRNA target predictions were made in flies by taking into account the genomes of several different insect species [67,70]. Out of around 10,000 unique genes in *Drosophila melanogaster*, at least 15% are predicted to be regulated by at least one miRNA; one fifth of these targets could be subject to coordinate control by two or more miRNAs from different families. In mammals, from 30 to 50% of total mRNAs appear to be targeted by miRNAs, although this last point is still controversial [33,48,71,72]. It should be kept in mind that only a small number of predictions have actually been biologically validated.

A full list of miRNA targets should not only describe specific mRNAs, but how the entire group of targets regulates some biological function. For example, several miRNAs (let-7b, miR-30, miR-98, miR-103, and miR-135) whose expression is induced during neuronal differentiation of embryonic carcinoma cells are predicted to target preferentially neurogenesis-associated mRNAs [73], and a list of miR-124 targets is rich in mRNAs for RNA binding proteins [59]. Frequently, potential miRNA targets are found to be regulatory genes themselves, like transcription factors, RNA binding proteins and post-translational modifiers. For instance, *Drosophila *target genes annotated as transcription or translation factors are detected twice and four times more frequently, respectively, than expected by chance. Clearly, the combined action of miRNAs and transcription factors is able to tune protein expression in a manner that cannot be achieved by transcription factors alone. For instance, human cell granulopoiesis is controlled by a mini circuit including miR-223 and the two transcription factors NF1-A and C/EBPalpha [74]. Analysis of the functional annotations of target genes in *Drosophila *indicates that miRNAs regulate a large variety of genes in many different biological processes. These potential targets are rich in genes that are expressed at specific developmental stages and that are involved in cell fate specification, morphogenesis and the coordination of developmental processes, as well as genes that are active in mature nervous system.

### MicroRNA in brain

Many miRNAs are highly conserved in different organisms, while others appear to readily enter and exit the genome, or to be created in viruses. The miRNA database at Sanger Center [75] lists over three thousand eight hundred unique mature miRNA sequences from different species: 462 are of human origin and a large fraction is present in brain at different levels [73,76-86]. About 20%–40% of miRNAs in brain appear to be developmentally regulated; the expression of certain families increases dramatically in parallel with cortical development [73,82,86]. The events that characterize neuronal differentiation and synapse formation are clearly associated with a distinct miRNA profile [76]. Clear-cut partitions in miRNA expression are also detected in the course of primary neuron differentiation in culture, with some miRNAs (miR-124, miR-128) preferentially expressed in neurons, some (miR-23, miR-26, miR-29) restricted to or more strongly expressed in astrocytes, and some (miR-9, miR-125) evenly distributed between these cell types [87]. Other conditions in which specific miRNAs are expressed include retinoic acid induced neuronal differentiation of embryonic carcinoma cells, and stem cells undergoing neural differentiation [34,73,88].

Target gene predictions in *Drosophila *suggest that miRNAs in the nervous system may take part in cell fate decisions, neural connectivity, cell shape and adhesion, and synapse function. Many neural transcription factors appear to be targets [70]. A large number of predicted target genes are implicated in cell fate decisions in the course of development; many of the developmental factors are re-employed in the mature nervous system to control synaptic function [70]. Several presumptive miRNA target genes are components of the Notch pathway [89,90]. These include factors that bind to Notch or modify its sensitivity to ligands, and Notch signalling target genes like *hairy *and several transcriptional regulators of the E(spl) and Bearded complexes [91].

Genes in these two complexes have short (6–7 nucleotide) conserved motifs in their 3' UTRs, which are involved in post-transcriptional control and are complementary to *D. melanogaster *miRNAs (miR-2, -4, -5, -6, -7, -11 and -79) [90]. In addition, predicted miRNA targets include factors involved in the asymmetric cell division of neuroblasts and transcription factors regulating different aspects of neuronal differentiation. Many predicted miRNA targets are secreted and trans-membrane factors that mediate the growth and guidance of axons and dendrites, and the formation of synapses. All these factors are conserved and have similar axon guidance functions in vertebrates. In addition, *Drosophila *miRNAs appeared to hit the cellular machinery that effects cell shape change and adhesion, including regulators and components of the cytoskeleton and of the cell junctions. Additional miRNA target genes that are active in the mature nervous system include neurotransmitter receptors, ion channels and pumps, as well as factors involved in neurotransmitter transport and synaptic release. According to a computational analysis, however, only a small number of regulatory miRNA-mRNA relationships appeared to be conserved between flies and mammals [67].

### Neuronal differentiation

**Figure 3:**
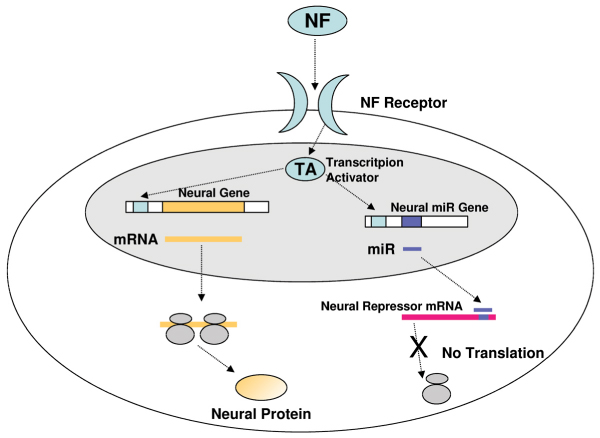
**Regulation of neuronal differentiation through miRNAs**. The neural factor NF, such as a neurotrophin (BDNF, NGF) or a neurotransmitter (glutamate, serotonin), interacts with its receptor, activating a signalling cascade that switches on transcription of selected neural genes, including specific miRNA genes as well. miRNAs, in turn, can inhibit translation of proteins regulating neuronal development, for instance a neural repressor protein. Such a regulated circuit of gene expression has been recently demonstrated for neuronal morphogenesis triggered by the cAMP-response element binding protein CREB [95]. This transcriptional activator has a central role in neural specific gene expression, being implicated in many neuronal processes like differentiation, plasticity, LPT, and memory formation. miR 132, whose transcription is activated by neurotrophins and is under direct CREB control, interacts with 3'UTR of the mRNA coding for the GTPase-activating protein p250GAP. This protein is a repressor of neuronal differentiation: its inhibition promotes neurite outgrowth.

Current research confirms a conserved role for miRNAs in proper development and maintenance of the central nervous system in mammals. miRNAs might act not only to change cell fate initially, but also to stabilize it at a later stage, by maintaining lineage-specific expression patterns. During differentiation, progenitor cells express families of miRNAs in a sequential manner [82], resulting in lineage-specific changes in the translation of subsets of transcripts. The great variety of neuronal cell types may utilize miRNAs to lock in a precise identity notwithstanding the gene fluctuations that normally occur in cells. This role may be most vital in cells derived from the same lineage that differ in only minute traits from each other. The global effect of given miRNAs on a set of targets may be to help establish a transcriptional profile characteristic of the cell in which the miRNAs are normally expressed. Tissue specific expression of miRNAs may therefore constitute a sort of "miRNA code", dictating in part the tissue-specific protein expression profile [92-95] (Figure 2). In parallel, the arrangement of miRNA binding sites in the 3' UTRs of mRNAs would constitute another code that could be read by the set of expressed miRNAs to tune product levels [96] (Figure 3). The translation of all transcripts that share a 3' UTR code would be regulated similarly. A possible example are the mRNAs targeted by miR-124a, one of the most abundant miRNAs in the brain. The binding motif for miR-124a is also one of the most prevalent miRNA binding sites in the 3'UTRs of mammalian transcripts [97]. Injection of miR-124a into HeLa, a human carcinoma cell line, cells caused the down-regulation of more than 100 messages and induced a new gene expression profile resembling that of a mature neuron [98]. Other studies have suggested that over-expression of the pro-neural transcription factors MASH1 and neurogenin promotes differentiation of neural progenitor cells into neurons, in part by inducing miR-124a expression [99]. The miRNA functions in cell differentiation and growth control, together with the location of some miRNA genes at sites of translocation or deletion breakpoints, has led to the idea that these molecules could play a relevant role in cancer development [55,100,101]. In the highly malignant brain tumor glioblastoma, the presence of markedly elevated levels of miR-21 may contribute to the malignant phenotype by acting as an anti-apoptotic factor [102].

### Synaptic plasticity

**Figure 4:**
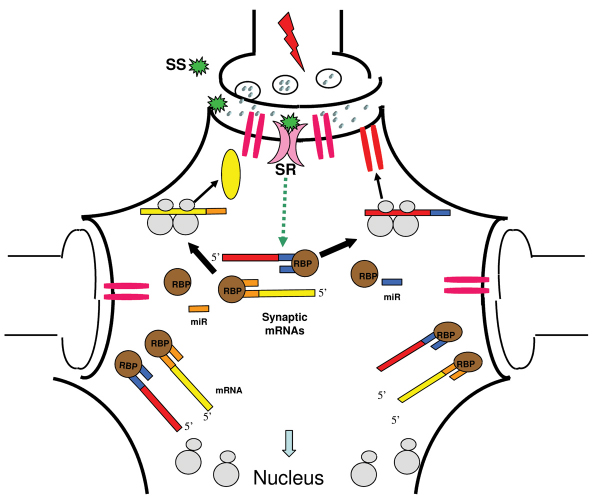
**Potential function of miRNAs in synaptic plasticity**. Three different synapses for a single neuron are shown. Only the central one is active, as it receives an electrical pulse, and is potentiated. In the inactive synapses, translation of synaptic mRNAs (red and yellow) is prevented by miRNAs (miR) interacting with their 3'UTRs, an interaction supposedly stabilized by RNA binding proteins (RBP). A synaptic stimulus SS, represented by a neurotrophin or a neurotransmitter (e. g. BDNF and glutamate, respectively), is sensed by a receptor, in pink (e.g. TrkB or the NMDA receptor), and transmitted inside the neuron, in the vicinity of the active synapse. This signals triggers dissociation of the mRNA:miRNA:RBP complex, allowing the local translation of synaptic mRNAs. The neo-synthesized proteins (e. g. an AMPA receptor, in red, or some other synaptic component, in yellow) are utilized at the active synapse, contributing to strengthen its response.

There are several reasons to believe that miRNAs may feature prominently in the function of the nervous system. Neuronal function is crucially determined by differential gene activity between and even within cell compartments, for example the different branches of a dendritic tree. The distances between nucleus and neurite projections are relatively long, making nuclear regulation of neurite properties problematic. As neuronal synapses are a site of active protein synthesis, translational control appears to be more efficient than transcriptional control for modulating gene activity near the site of action. Several miRNAs are found within the actively translating poly-ribosome fraction, where they might work as mediators of translational regulation [76,86]. Regulation by miRNAs would thus provide an excellent additional mechanism of post-transcriptional control of gene expression. In the case of axon guidance, it has been shown that the relative abundance of adhesion molecules and chemotropic receptors on the surface of the growth cone is post-transcriptionally regulated in response to external cues. Local control of protein synthesis in mammalian dendrites is essential for synaptic plasticity phenomena (e. g. LTP) that contribute to the molecular basis for learning and memory formation [103,104]. The brain derived neurotrophic factor BDNF, which is essential for establishing LTP at hippocampal synapses [104], regulates the local translation of a group of mRNAs [105]. miRNAs may have a role in modulating local translation in response to changes in synaptic activity, thus regulating synaptic strength and growth of spines [96,106]. According to this view, a set of miRNAs would inhibit the translation of associated mRNAs under basal conditions, with the plausible support of RNA binding protein(s). The inhibition would be released as a consequence of synaptic activity, leading to de-repression of a set of mRNAs for post-synaptic proteins capable of strengthening the synaptic response (Figure 4). One connection between miRNAs and the mechanism of translational inhibition might be supplied by RNA binding proteins, like FMRP, complexed with messenger ribonucleoproteins (mRNPs) and poly-ribosomes. A current model considers FMRP, or functionally similar RNA binding proteins, as a platform that localizes to appropriate dendritic sites a set of mRNAs, which encode the components required for enhancing synaptic response or growth. Such translation platforms may be turned on by carefully promoting the translation of critical mRNAs and turned off by directing miRNAs to mRNAs on the platform or to the mRNA encoding the platform protein(s) itself [34].

### miRNA syndromes?

It must not be surprising that miRNA biogenesis and function are implicated in neurological disease.

One example is Tourette's syndrome, a developmental neuropsychiatric disorder characterized by vocal and motor tics. Recent findings point to its association with rare sequence variants of the gene encoding the trans-membrane protein SLITRK1 (Slit and Trk-like 1), on chromosome 13q31.1 [107]. Wild-type SLITRK1 enhances dendritic growth in primary neuronal cultures. Variants influencing the binding site for human miR-189 in the 3'UTR region of the gene were found in some affected people but not in control chromosomes. Interestingly, SLITRK1 mRNA and human miR-189 showed an overlapping expression pattern in brain regions implicated in Tourettes syndrome.

A second case is DiGeorge syndrome, a frequent disease characterized by a variety of defects, including schizophrenia and obsessive-compulsive disorder. The gene for the double-stranded RNA-binding protein DGCR8, involved in miRNA processing as an essential Drosha co-factor, maps in a region in chromosome 22 (22q11.2) which is commonly deleted in people affected by the disease [101,108]. This finding suggests that defects in miRNA biogenesis may contribute to the widespread developmental abnormalities affecting DiGeorge syndrome patients.

The third example is Fragile × syndrome, the most common inherited cause of human mental retardation. The disease is most often caused by a trinucleotide (CGG) repeat expansion in the fragile × mental retardation 1 gene, encoding the RNA-binding protein FMRP, which is found in neurons and transported into dendrites. FMRP is involved in transport and localization of a subset of neuronal mRNAs as well as in regulating their translation, and appears to play an important role in dendritic spine maturation or plasticity [109]. FMRP associates with miRNAs in both *Drosophila *and mammals, indicating that it may regulate translation via the miRNA pathway [110]. miRNAs could function similarly to BC1 noncoding dendritic RNA to determine the specificity of FMRP function by linking the regulated mRNAs to FMRP [23].

## Conclusion

Despite the fact that non coding RNAs in brain have been characterized only recently, a large amount of experimental data indicate their importance in brain development and function. They clearly seem to be involved in all mechanisms of synaptic plasticity like memory and stress response. Furthermore, anomalous non coding RNA expression has been linked to several brain dysfunctions in humans, suggesting their possible targeting for therapeutic treatment or their use as therapeutic tools.

## Abbreviations

3'UTR: 3' Untranslated Region; 5-HT_2C_: Serotonin 2C Receptor; BDNF: Brain Derived Neurotrophic Factor; FMRP: fragile × syndrome protein; LNA: Locked Nucleotide RNA; mRNA: messenger RNA; mRNP: messenger ribonucleoprotein; miRNAs: microRNA; ncRNA: non coding RNA; NGF: Nerve Growth Factor; PABP: Poly-A Binding Protein; PCR: Polymerase Chain Reaction; pre-miRNA: microRNA precursor; pri-miRNA: microRNA primary transcript; PWS: Prader-Willi Sindrome; RISC: RNA-induced Silencing Complex; RNP: Ribonucleoprotein Particle; rRNA: ribosomal RNA; SINE: Short Interpersed Repetitive Element; SLITRK1: Slit and Trk-like 1; snoRNA: small nucleolar RNA; snoRNP: small ribonucleoprotein particle; snRNA: small nuclear RNA; tRNA: transfer RNA

## Acknowledgements

S.N. acknowledges financial support from MIUR FIRS project "Neurobiotechnologies".

This article has been published as part of *BMC Neuroscience* Volume 7, Supplement 1, 2006: Problems and tools in the systems biology of the neuronal cell.  The full contents of the supplement are available online at .
